# Feasibility and Acceptability of an Online WhatsApp Support Group on Breastfeeding: Protocol for a Randomized Controlled Trial

**DOI:** 10.2196/32338

**Published:** 2022-03-09

**Authors:** Kris YW Lok, Rachel WT Ko, Heidi SL Fan, PH Chau, Janet YH Wong, MP Wang, Vicky Tsang

**Affiliations:** 1 School of Nursing, Li Ka Shing Faculty of Medicine University of Hong Kong Pokfulam Hong Kong; 2 Natural Parenting Network Hong Kong Hong Kong

**Keywords:** mHealth, breastfeeding, peer support, mobile health, parenting, instant messaging, online support, women's health, postpartum health, postpartum support

## Abstract

**Background:**

Mobile health, the use of mobile technology in delivering health care, has been found to be effective in changing health behaviors, including improving breastfeeding practices in postpartum women. With the widespread use of smartphones and instant messaging apps in Hong Kong, instant messaging groups could be a useful channel for delivering breastfeeding peer support.

**Objective:**

The aim of this paper is to study the feasibility and acceptability of an online instant messaging peer support group by trained peer counselors on improving breastfeeding outcome in primiparous women in Hong Kong.

**Methods:**

A two-arm, assessor-blind, randomized controlled feasibility study will be conducted on 40 primiparous women with the intention to breastfeed. Participants are recruited from the antenatal obstetrics and gynecology clinic of a public hospital in Hong Kong and randomly assigned at a 1:1 ratio to either intervention or control group. The intervention group receives peer support in an online instant messaging group with trained peer counselors on top of standard care, whereas the control group receives standard care. Breastfeeding outcome will be assessed for 6 months post partum or until weaned. The breastfeeding status, the proportion and duration of exclusive and any breastfeeding in each group, and the self-efficacy and attitude of participants will be assessed. The feasibility and acceptability of the study would also be assessed in preparation for a full randomized controlled trial.

**Results:**

This study (protocol version 1 dated January 5, 2021) has been reviewed and approved by the institutional review board of the University of Hong Kong, Hospital Authority Hong Kong West Cluster (reference UW 21-039), on January 26, 2021. Data collection is ongoing and expected to be completed in December 2021. The findings will be updated on clinical trial registry and disseminated in peer-reviewed journals.

**Conclusions:**

This study aims to assess the feasibility and effectiveness of an online instant messaging peer support group in improving the breastfeeding outcome of primiparous women in Hong Kong. Its findings could inform the feasibility of a full-scale trial with this intervention design.

**Trial Registration:**

ClinicalTrials.gov NCT04826796; https://clinicaltrials.gov/ct2/show/NCT04826796

**International Registered Report Identifier (IRRID):**

DERR1-10.2196/32338

## Introduction

Mobile health (mHealth) is the delivery of medical and public health practices using mobile devices [1]. With the advances and increasing ubiquity in mobile technologies, there is an increase in the use of mHealth interventions for promoting healthy infant feeding practices and breastfeeding. A recent meta-analysis has reported that mHealth significantly improved exclusive breastfeeding initiation, breastfeeding attitude, and knowledge [2]. However, the effectiveness of the more interactive and adaptive platforms, such as smartphone apps and social networking tools, in improving breastfeeding outcomes remains understudied.

Smartphones and social networking apps have emerged in recent years as the new tool for information acquisition and exchanges. In 2019, the number of smartphone users in Hong Kong was estimated to reach 6.5 million. It is predicted that 93% of the population in Hong Kong will be smartphone users by 2025 [3]. Previous studies have found the use of these online discussion and social networking platforms to be an acceptable, affordable, and accessible method for a range of health promotion intervention, including promoting physical activities [4], healthy diet [5], smoking cessation [6], and reducing alcohol use [7]. As these communication tools allow for more personalized and instant response without time and location limitations, interventions delivered through online social networking platforms tend to have a high uptake. These platforms could be a promising tool for breastfeeding promotion. A recent integrated review has found that women tend to seek online breastfeeding support if they were isolated or do not receive sufficient professional support, and that online support could be an accessible and easily available channel for them to learn from others’ experience [8]. Nevertheless, most studies on the topic to date have focused on the qualitative aspects of the online support experience [8], and there remains a lack of intervention studies on online peer support intervention. Moreover, existing data on whether online breastfeeding peer support could help improve breastfeeding outcome remain scarce and inconclusive [8].

According to the most recent survey, more than 87.5% of women initiated breastfeeding in Hong Kong in 2018 [9]. However, half of the breastfeeding women have never exclusively breastfed their babies [9]. The exclusive breastfeeding rate tailed off during the first 2 months and dropped to 26.3% at 6 months [9]. Considering the importance of exclusive breastfeeding in the first 6 months, this low exclusive breastfeeding rate is of public health concern. While practices vary, many women in Hong Kong continued to adopt postnatal rituals that Chinese women adhere to in the first month post partum, such as staying at home, eating prescribed foods (eg, chicken and Chinese ginger), and avoiding bathing or washing their hair. This would consequently mean that Chinese women would stay homebound for 1 month after giving birth and often follow specific restrictions on their diets and activities. The existing antenatal and postnatal breastfeeding support is mainly provided by hospitals and maternal and child health centers, which due to the pandemic, have been further limited. Postpartum women in Hong Kong, thus, may face additional challenges in seeking timely breastfeeding support. Unresolved problems and inadequate support are risk factors for women to cease exclusive breastfeeding early [10,11]. There is a need for innovative approaches to engage and support breastfeeding women in the first 6 months after childbirth.

Thus, a randomized controlled feasibility study is proposed with the aim to examine the practicality and feasibility of supporting Chinese breastfeeding mothers in Hong Kong. In a previous study conducted in Hong Kong, it is suggested that peer support delivered via instant text messages could be a viable way of promoting breastfeeding [12]. The instant messaging smartphone app, WhatsApp (WhatsApp Inc), is one of the most used social networking platforms in Hong Kong [13]. It is a free all-in-one app for sending text and voice messages, multimedia contents, and video calls. The proposed intervention aims to address an important service gap in Hong Kong to promote and sustain exclusive breastfeeding. While WhatsApp has been used as a tool to deliver professional breastfeeding support in countries such as Turkey [14], this will be the first WhatsApp breastfeeding support intervention provided by trained peer supporters in Hong Kong. However, there might be potential barriers to a successful implementation of the intervention and evaluation of its effectiveness. Therefore, a feasibility pilot study is proposed to identify if a WhatsApp online group on breastfeeding by peer counselors, delivered through instant messaging support and designed to improve breastfeeding outcomes, is feasible and acceptable.

This trial was registered on ClinicalTrials.gov (NCT04826796) on April 1, 2021.

## Methods

### Study Aim

The main objective of the study is to determine the feasibility and acceptability of an online messaging support group hosted by trained peer counselors on breastfeeding outcome in women with intention to breastfeed in Hong Kong over a 6-month period.

### Trial Design

This trial adopts a randomized controlled superiority design with 2 parallel groups and a primary end point of cessation of breastfeeding in the first 6 months post partum. Participants will be randomly allocated to the intervention or control group on a 1:1 ratio.

### Study Setting

Participant recruitment will be conducted at the obstetrics and gynecology outpatient clinic at a public hospital in Hong Kong serving an urban population.

### Sample Size

The sample size of the trial is 40 participants, with 20 participants in each arm. As this is a feasibility and pilot study that aims to gauge the rate of recruitment, adherence, and retention levels, as well as to identify unanticipated issues of the trial design, this recommended sample size for feasibility and pilot study is used [15]. Recruitment will continue until target sample size is reached.

### Inclusion and Exclusion Criteria

To be eligible for inclusion in the study, participants must (1) be primiparous, (2) intend to breastfeed, (3) have a singleton pregnancy, (4) have term infant (37-42 weeks gestational), (5) be Cantonese speakers, (6) reside in Hong Kong, and (7) have no serious medical or obstetrical complications.

Individuals will be excluded from the study if they fail to meet the inclusion criteria or if their newborn (1) are <37 week gestation; (2) have an Apgar score <8 at 5 minutes; (3) have a birthweight <2500 grams; (4) have any severe medical conditions or congenital malformations; (5) are placed in the special care baby unit for more than 48 hours after birth; or (6) are placed in the neonatal intensive care unit at any time after birth. Participants who are, due to mental or physical reasons, unable to provide written informed consent are also excluded from the study.

### Recruitment, Randomization, and Allocation of Intervention

Recruitment will be conducted at the obstetrics and gynecology outpatient antenatal clinics in a public hospital in Hong Kong. Research assistants will have obtained written informed consent from participants. All eligible participants who consented to participate will be randomized. They will be randomly assigned to either the intervention or control group at a 1:1 ratio. The randomization sequence was computer generated using STATA (Stata Corp) with simple randomization by a researcher prior to study recruitment. The sequence will be concealed in a password-encoded excel file, which will not be assessed by nor disclosed to a second researcher responsible for participant recruitment and data collection. After the participants are recruited and the baseline assessments have been completed, a third researcher with access to the randomized sequence will notify the participants of their group assignment.

Due to the nature of the study, a single-blind design is used. The participants will be notified of their group assignment after the completion of baseline assessment. The researcher responsible for study recruitment and assessments will be blinded to group allocation.

### Intervention

The participant timeline in the study is outlined in Table 1. The control group will continue to receive standard care, whereas the intervention group will, in addition to standard care, be included in a breastfeeding support group on a popular online messaging mobile app, WhatsApp (“WhatsApp group”), with trained peer counselors. The participants will be notified before they were added to the group and will receive a standard welcome message in the group afterward. The message will introduce the peer counselors and encourage participants to raise any question or concern they may have. Peer counselors will send prompts asking for questions or send information related to breastfeeding every week for 6 months. No harm to the participants is anticipated. The participants could leave the WhatsApp group on their own if they wish to discontinue the intervention. The participants are permitted to receive other professional breastfeeding support.

**Table 1:** Participant timeline in the WhatsApp breastfeeding peer support study.

Point of contact		Study Period					
		Enrollment	Allocation	Postallocation			
		Study entry	Allocation	1 month post partum	2 months post partum	3 months post partum	6 months post partum
**Enrollment**							
	Eligibility screen	✓					
	Informed consent	✓					
	Allocation		✓				
**Interventions**							
	Control group			Standard care			
	Intervention group			WhatsApp peer support group and standard care			
**Assessments**							
	Demographics	✓					
	Infant feeding status			✓	✓	✓	✓
	Breastfeeding self-efficacy	✓			✓		
	Breastfeeding attitude	✓			✓		

In this trial, the WhatsApp group includes 4 trained peer counselors, 1 of whom is an experienced trainer, and all participants are allocated to the intervention group. The trained peer counselors are all women living in Hong Kong with at least 4 months of breastfeeding experience. All of them have been trained under the Breastfeeding Peer Support Scheme organized by Natural Parenting Network and have received at least 16 hours of training, attended 2 practicums, and passed a standardized assessment. The training program covered topics including (1) why breastfeeding is important, (2) how to assure good start of breastfeeding, (3) how to help mother breastfeeding, (4) communication skills, (5) common breastfeeding problems, (6) diet and hygiene, (7) maternal illness and needs, and (8) local support and role of peer counselors. The peer counselors were assessed by their trainers after completing the training program.

### Outcomes

The feasibility of the study will be examined based on the proportion of participants agreed to participate in the study and the acceptance of women to be randomized; completion of the intervention and follow-up at 6 months post partum will also be measured. Data on the number of women approached and screened and the reasons for rejection or exclusion will be collected. In addition, the women’s views on the intervention, including strengths, weaknesses, and room for improvements, will be assessed at each follow-up time points. The reason for dropping out of intervention would be recorded. Women in the intervention group will also be invited to a qualitative interview assessing their views on the intervention at the end of the study.

In terms of intervention efficacy, the primary outcome of the study is the infant feeding status. We will collect data on the participants’ infant feeding status at 1, 2, 4, and 6 months post partum. The infant feeding status will be classified according to the World Health Organization definitions in the 24 hours prior to each data collection period [16]. We will compare the proportion of any and exclusive breastfeeding among participants in the intervention and control groups. The secondary outcomes are the breastfeeding self-efficacy and attitude of the participants. Breastfeeding self-efficacy will be measured using the Hong Kong Chinese version of the Breastfeeding Self-Efficacy Scale-Short Form (BSES-SF) [17]. BSES-SF is a 14-item scale with total score ranging from 14 to 70, with a higher score indicating higher breastfeeding self-efficacy [18]. Attitude toward breastfeeding will be measured using the Chinese version of the 17-item Iowa Infant Feeding Attitude Scale (IIFAS) [19]. The total score ranges from 17 to 85, with a higher score indicating a more favorable attitude toward breastfeeding [20]. The breastfeeding self-efficacy and attitude of participants will be measured at study entry and 2 months post partum. All participants will be followed for 6 months post partum or until weaned.

### Data Collection Method

Demographic information and breastfeeding self-efficacy and attitude will be collected from the participants at study entry. The participants will be asked to complete the questionnaires after providing informed consent to participate. Demographic information such as age, educational level, household income, intention to exclusively breastfeed, and intended duration of any and exclusive breastfeeding, whether they were breastfed as a child, and whether they know someone with breastfeeding experience, were collected. Maternal and birth data will be collected by the research assistant from the participants’ medical records. Follow-up assessments will be conducted via telephone interviews at 1, 2, 4, and 6 months post partum by the research assistant. To promote retention, the participants will be asked their preferred time of contact for the phone interviews. Infant feeding status will be assessed at all 4 follow-up time points, whereas the breastfeeding self-efficacy and attitude will be assessed at 2 months post partum. Any professional breastfeeding support received will also be recorded.

### Data Management Plan

Data containing personal information will be stored separately in a locked cabinet at the School of Nursing or on password-encoded files. Data entry will be completed by the research assistants. Range check for data value will be conducted to promote data quality. Only study investigators and research assistants will have access to the data set.

### Data Analysis

To assess the feasibility of the study, descriptive statistics of the proportion of individuals who agreed to participate and to be randomized and who completed the study, as well as any loss to follow-up will be reported. The main reasons for rejection and exclusion will also be reported. To compare the baseline characteristics of the participants across the 2 study groups, *t* tests will be used for continuous variables and chi-square tests for categorical variables. Moreover, we will compare and report the breastfeeding outcomes in the 2 study groups and will check for evidence of harm. Intention-to-treat analysis will also be conducted. Multiple imputation will be used to account for missing data, and reporting will follow the guideline published in the British Medical Journal [21]. Where appropriate, each estimate will be accompanied by a 95% confidence interval, and a 5% level of significance will be used in all statistical tests. Data analysis will be performed using the Stata (version 16.0) statistical software [22].

### Data Monitoring

As the intervention poses minimal risks to the participants, no data monitoring committee is needed. However, the dialogue and responses from the peer supporter and participants are recorded and monitored in the intervention to ensure that queries are being responded to appropriately, and to ensure that any negativity that may be generated by difficulties experienced by new mothers is handled skillfully. Investigators will have the final decision to terminate the trial if needed. Any serious adverse events will be reported to the ethics committee within 1 week, and trial record will be made available for audit by the ethics committee.

### Ethics Approval

This study has been reviewed and approved by the institutional review board of the University of Hong Kong, Hospital Authority Hong Kong West Cluster (Reference UW 21-039) on January 26, 2021. Any amendment to the protocol will be submitted to the institutional review board for review and approval. This study complies with the Declaration of Helsinki and its later amendments. All participants have provided written informed consent to participate.

## Results

Study recruitment has commenced in March 2021. Data collection is ongoing. The projected end date of intervention and data collection is at the end of December 2021. The result from this study will be updated on the clinical trial registry and submitted to suitable peer-reviewed publications within 12 months of study completion. Anonymized data will be available upon reasonable request within 24 months of study completion.

## Discussion

This protocol for a randomized controlled trial aims to study the feasibility and potential efficacy of a WhatsApp group with trained peer counselors in improving breastfeeding outcomes in primiparous women in Hong Kong. The small sample size may limit the power and generalizability of the study; however, as a feasibility study, this could provide valuable information for conducting a full-scale randomized controlled trial.

## Abbreviations

BSES-SF: Breastfeeding Self-Efficacy Scale-Short Form
IIFAS: Iowa Infant Feeding Attitude Scale
mHealth: mobile health
